# Food‐related allergic reactions in a school setting with a strict management plan

**DOI:** 10.1111/pai.70172

**Published:** 2025-08-12

**Authors:** Alexandre Piletta‐Zanin, Alexander Scherl, Didier Ortelli, Patrick Edder, Philippe A. Eigenmann

**Affiliations:** ^1^ Pediatric Allergy Unit, Department of Woman, Child and Adolescent University Hospitals of Geneva (HUG) Geneva Switzerland; ^2^ Official Food Control Authority of the State of Geneva (SCAV), Health Department Geneva Switzerland

**Keywords:** food allergy, individualized care plan, reaction, real‐life, school restaurant

To the Editor,

Food allergy (FA) is the main cause of anaphylaxis in children, and approximately 3%–8% of school‐aged children have food allergy.^1^ Symptoms of FA can vary greatly and can range from food oral allergy syndrome (OAS) to severe anaphylaxis.^2^

As the prevalence of food allergy among school‐aged children is increasing, prevention of reactions in schools becomes an important public health concern. Moreover, after reports of severe anaphylaxis in school settings, including deaths, school policies have become stricter and awareness of anaphylaxis management has been raised.^2^

The management of food allergies in schools includes strategies to minimize the risk of allergen exposure, as well as procedures for recognizing and adequately treating allergic reactions and anaphylaxis.^3^ Appropriate medical information to the school about the child's potentially severe food allergy with a written Individualized Care Plan (ICP) or food‐allergy action plan is the most used approach.^3^ Another strategy has been to ban certain foods in school settings, with peanut‐free schools being implemented in the United States; however, without reducing the number of reactions and the use of epinephrine.^4^

It remains undefined how effective specific strategies for avoiding accidental reactions in children with food allergies in the school setting are. To address this issue, we went through the data of our recently published study, which consisted of correlating the severity of food allergy in real‐life conditions to the amount of allergen consumed. Among the various recruitment strategies, we systematically and prospectively recruited all subjects in the State of Geneva who experienced an allergic reaction to food in school restaurants over the course of one school year.^5^ For this focused sub‐analysis, we specifically extracted all reactions that occurred in the school restaurant setting.

In Geneva, it is compulsory in the school setting for a child with a food allergy to establish an ICP specifying the foods to avoid and the medical procedure to apply in case of an allergic reaction.^6^

If the food allergy requires a simple diet (e.g., food items that are visually recognizable, consumed in their unprocessed form, and easily removed from a prepared meal), the staff will ensure the exclusion of the allergenic food without requiring a special meal or allowing legal guardians to supplement the meals provided by the school restaurant.^6^ If the food allergy requires a more complex diet (e.g., common allergens hidden in processed foods such as eggs or nuts) and involves the use of an anaphylactic treatment (adrenaline auto‐injector), the legal guardians must provide packed meals for lunch and/or snacks.^6^

For recruitment of patients with an allergic reaction in school restaurant settings, the main challenge was to inform all the caregivers working in school restaurants about the study. We acted through two main channels of information: an email with information on the study to all collaborators sent by the general manager and a poster located in all facilities welcoming children for meals. An email was sent 1 month before the start, on the starting day, and every 3 months to remind supervisors. The email and poster contained details on [1] how to recognize allergic symptoms, [2] how to keep food samples, and [3] how to contact the study team.

In the State of Geneva with a population of 500,000 inhabitants, school restaurants provide every day a lunch to an average of 17,522 children in 143 different premises, with a supervisor for 12.5 children. Within this population, 572 children diagnosed with a food allergy receive a packed meal provided by their parents.

With 1'734'678 meals served during 1 year, we were notified of 14 food allergic reactions in children between 5 and 11 years old (median = 9 years). The number of reactions was confirmed with a separate listing provided by the head manager of school restaurants. Among the 14 reactions, 2 were immediate‐type allergic reactions to non‐cross‐reactive foods, and 9 were related to OAS. Three reactions could not be properly identified. Most of the children (13/14) had a first‐time reaction and did not have an ICP. One accidental reaction consisted of a patient with an ICP to goat milk who was given by error feta cheese (goat cheese) by a caregiver and who presented a moderate skin reaction.^7^ The other immediate non‐cross‐reactive reaction was a severe reaction (facial edema, vomiting, and loss of consciousness) after a first consumption of hazelnut, which required paramedic care and adrenaline use. OAS was most frequent to pineapple^3^ and kiwi.^2^ These two fruits have been previously mentioned to be a main source of oral food allergy syndrome.^8^ Details of the reactions are outlined in Table 1.

**TABLE 1:** Details on reactions in school settings.

Age (years)	Gender	Eliciting food	Symptoms	Management
5	F	Pineapple	Oral pruritus	Spontaneous resolution
5	M	Pineapple	Oral pruritus	Spontaneous resolution
5	F	Fruit compote	Oral pruritus	Spontaneous resolution
5	M	Lobster pasta	Skin rash	Spontaneous resolution
6	M	Raw peach	Oral pruritus	Spontaneous resolution
7	M	Kiwi	Oral pruritus	Unknown
9	M	Pineapple	Oral pruritus	Spontaneous resolution
9	F	Egg custard	Urticaria	Oral antihistamine
9	F	Canelloni	Oral pruritus	Spontaneous resolution
9	M	Goat milk in ravioli	Skin rash	Oral antihistamine
9	F	Cherry pie	Oral pruritus	Spontaneous resolution
9	F	Kiwi	Oral pruritus	Spontaneous resolution
10	F	Hazelnut in pastry	Facial edema, vomiting, and loss of consciousness	i.m. adrenaline by paramedics
11	M	Cauliflower, rice, fruits	Skin rash	Spontaneous resolution

Several conclusions arise from these observations. [1] A strict school policy with lunch provided from home and a mandatory ICP result in a low annual incidence of allergic reaction (incidence = 1 reaction/123′905 meals). However, the annual incidence of adrenaline use (0.57/10′000 children or 1/1′734′678 meals) is similar to that previously reported, as some studies report use of adrenalin at a rate of 0.2–0.6/10'000 children.^4^ Nevertheless, the cost for this is a time‐consuming procedure and a heavy burden on the family. [2] Even very strict school policies cannot totally prevent allergic reaction, as first‐time reactions can occur in all settings. In schools, it is estimated that 25% of anaphylaxis occurs in patients with undiagnosed allergy,^9^ which in our study was the case for all but one child. [3] Most of the reactions in our study were related to OAS, with a very low risk of anaphylaxis. Indeed, OAS is very common in school‐age children, as it has been previously reported that 7%–14% of school‐age children present OAS.^8^, ^10^ If wrongly identified, OAS can lead to anxiety in caregivers and unnecessarily prevent children from eating regular meals in school restaurants.

There are several limitations to this report. First, the initial study was not designed specifically to examine reactions in schools. Secondly, several reactions could have been missing, either because they were not notified by the care givers or because the reaction was not reported to us. This risk has been minimized by the dual reporting system. Finally, the low number of reactions prevents us from drawing definite conclusions.

Our study highlights the difficulty for school restaurants to find a good balance between preventing severe allergic reactions and having too strict restriction measures. Also, the collaboration between schools, families, and allergists is of great importance to correctly identify patients at risk of presenting severe allergies from patients having OAS. In the last decades, restrictions in school restaurants have constantly increased, which probably effectively prevented numbers of allergic reactions, but with a clear added burden for the families and the caregivers. Data such as the one provided in our study are important for providing an appropriate balance in preventive measures.

## AUTHOR CONTRIBUTIONS

**Alexandre Piletta‐Zanin:** Investigation; conceptualization; writing – review and editing; writing – original draft. **Alexander Scherl:** Formal analysis. **Didier Ortelli:** Formal analysis. **Patrick Edder:** Formal analysis. **Philippe A. Eigenmann:** Conceptualization; writing – review and editing.

## FUNDING INFORMATION

State of Geneva, University Hospitals of Geneva.

## CONFLICT OF INTEREST STATEMENT

The authors report no potential conflict of interest.

## PEER REVIEW

The peer review history for this article is available at https://www.webofscience.com/api/gateway/wos/peer‐review/10.1111/pai.70172.
